# Note on the Subsequent History of a Case of Pylorectomy

**Published:** 1904-03

**Authors:** A. W. Prichard

**Affiliations:** Surgeon to the Bristol Royal Infirmary


### NOTE ON THE SUBSEQUENT HISTORY OF A CASE OF PYLORECTOMY.

BY

A. W. Prichard,

*Surgeon to the Bristol Royal Infirmary.*

---

In May, 1902, I read notes on a case of pylorectomy before the Society, which are reported in vol. xx., p. 285, of the *Medico-Chirurgical Journal*, 1902, and the case is given *in extenso* in the *Lancet* of August 23rd, 1902.

The patient recovered, and went on very well for sixteen months, having resumed her duties as cook, and complaining of nothing except occasional attacks of constipation, one of which was rather serious, but it yielded to aperients and enemata. She was able to eat and digest ordinary food, and gained flesh and grew quite fat.

On September 7th, 1903, she was re-admitted to the Infirmary complaining of pain on the right side of the abdomen, and she was looking ill. There was constipation, some distension of the abdomen, and tenderness. The case looked like one of appendicitis, but under the anæsthetic resistance could be felt right across the abdomen. I made a median incision, and could feel a mass filling the pelvis. On investigating this with my finger, pus in considerable quantity welled up from the region of the appendix. So I put a drainage tube through the parietes in the right flank and another in the posterior vaginal fornix. The patient never rallied, and died unrelieved three days after the operation.

At the autopsy there were many adhesions, but little fluid was found in the abdominal cavity. These adhesions were especially marked in the region of the pyloric end of the stomach, where there was new growth stretching along the lesser curvature. The lower coils of intestine were adherent, and there was across the pelvis a sarcomatous growth involving both ovaries. In the front part of the right ovary and adjacent to the cæcum was an abscess cavity emptied of pus. The cæcum was infiltrated with sarcoma. The appendix was normal.

The specimens shown (at the November meeting, 1903) were *(a)* The original pyloric growth removed in April, 1902; *(b)* The stomach and duodenum, showing the union of the latter to the shortened stomach; *(c)* The cæcum infiltrated with sarcoma; *(d)* The ovaries and uterus.

### Notes on the Examination of the Parts Removed, By Dr. E. H. Edwards Stack.

*Sections of growth removed by Operation.*—On longitudinal section the growth is seen to invade the whole circumference of the pylorus, extending about an inch and a half along the stomach wall, where it tapers off in the normal wall, and about half an inch towards the duodenum, where the edge comes to a more abrupt termination. The muscular coats are seen to be very much hypertrophied, and show up as clear strata amidst the opaque, tough white mass of the rest of the growth; no superficial ulceration can be made out; the lumen of the pylorus is narrowed to about the size of a lead pencil.

Microscopically the growth varies in different parts. In some places the whole section shows tough, fibrous tissue, with a great tendency to curl in strands like elastic tissue; in others there are here and there amongst the masses of cells some round, some oval, and some oat-shaped—these are larger and more definitely spindle-shaped than inflammatory cells could be. These masses extend close up to the bases of the glands. The glands are well shown in active state, the cells being much swollen and granular, and in places large "goblet" cells are present. The specimen is therefore one of sarcoma of the pylorus.

Section of the recurrence in the stomach shows that it is sarcoma, and without the excess of fibrous tissue shown in the original tumour.

Section of the ovary shows ordinary round and spindle-celled sarcoma.

---

### MORTALITY AND SICKNESS IN THE CITY OF BRISTOL FOR THE YEAR 1903.

